# Supplementary material for: Modeling glioblastoma heterogeneity as a dynamic network of cell states
Source: Mol Syst Biol. 2021 Sep 16;17(9):e10105. doi: 10.15252/msb.202010105 (PMC8444284; doi:10.15252/msb.202010105)
Supplement: Supplementary file 5 — Source Data for Figure 3 [file MSB-17-e10105-s001.zip › Figure3A_sourcedata/GSEA_3065/hallmarks_state5.GseaPreranked.1623416302988/HALLMARK_HEDGEHOG_SIGNALING.html]

Details for gene set HALLMARK\_HEDGEHOG\_SIGNALING[GSEA]

|  || Dataset | state5 |
| Phenotype | NoPhenotypeAvailable |
| Upregulated in class | na\_pos |
| GeneSet | HALLMARK\_HEDGEHOG\_SIGNALING |
| Enrichment Score (ES) | 0.4210158 |
| Normalized Enrichment Score (NES) | 1.2697453 |
| Nominal p-value | 0.13725491 |
| FDR q-value | 0.1617958 |
| FWER p-Value | 0.6 |
Table: GSEA Results Summary

  

Fig 1: Enrichment plot: HALLMARK\_HEDGEHOG\_SIGNALING      
 Profile of the Running ES Score & Positions of GeneSet Members on the Rank Ordered List

  

| PROBE | GENE SYMBOL | GENE\_TITLE | RANK IN GENE LIST | RANK METRIC SCORE | RUNNING ES | CORE ENRICHMENT || 1 | ADGRG1 |  |  | 74 | 0.567 | 0.1233 | Yes |
| 2 | SCG2 |  |  | 166 | 0.470 | 0.2223 | Yes |
| 3 | NRP2 |  |  | 319 | 0.374 | 0.2922 | Yes |
| 4 | THY1 |  |  | 546 | 0.299 | 0.3363 | Yes |
| 5 | NRCAM |  |  | 748 | 0.251 | 0.3721 | Yes |
| 6 | CELSR1 |  |  | 1257 | 0.178 | 0.3567 | Yes |
| 7 | OPHN1 |  |  | 1329 | 0.168 | 0.3879 | Yes |
| 8 | PTCH1 |  |  | 1526 | 0.150 | 0.4007 | Yes |
| 9 | VEGFA |  |  | 1634 | 0.139 | 0.4210 | Yes |
| 10 | NF1 |  |  | 2105 | 0.105 | 0.3929 | No |
| 11 | LDB1 |  |  | 2309 | 0.091 | 0.3915 | No |
| 12 | NRP1 |  |  | 3735 | 0.019 | 0.2368 | No |
| 13 | VLDLR |  |  | 3854 | 0.014 | 0.2268 | No |
| 14 | PML |  |  | 3947 | 0.009 | 0.2186 | No |
| 15 | TLE3 |  |  | 4004 | 0.007 | 0.2140 | No |
| 16 | RTN1 |  |  | 4556 | -0.016 | 0.1563 | No |
| 17 | RASA1 |  |  | 4642 | -0.020 | 0.1514 | No |
| 18 | ETS2 |  |  | 6076 | -0.078 | 0.0096 | No |
| 19 | AMOT |  |  | 6293 | -0.090 | 0.0064 | No |
| 20 | TLE1 |  |  | 7005 | -0.137 | -0.0410 | No |
| 21 | MYH9 |  |  | 7248 | -0.161 | -0.0306 | No |
| 22 | CRMP1 |  |  | 7368 | -0.177 | -0.0028 | No |
| 23 | HEY1 |  |  | 7370 | -0.177 | 0.0382 | No |
| 24 | DPYSL2 |  |  | 8121 | -0.301 | 0.0243 | No |
| 25 | CDK6 |  |  | 8156 | -0.310 | 0.0924 | No |
Table: GSEA details [plain text format]

  

Fig 2: HALLMARK\_HEDGEHOG\_SIGNALING: Random ES distribution      
 Gene set null distribution of ES for **HALLMARK\_HEDGEHOG\_SIGNALING**
